# (Furan-2-yl)(5-hy­droxy-3-methyl-5-phenyl-4,5-dihydro-1*H*-pyrazol-1-yl)methanone

**DOI:** 10.1107/S1600536811000948

**Published:** 2011-01-12

**Authors:** Hadi Kargar, Reza Kia, Majid Moghadamm, Muhammad Nawaz Tahir

**Affiliations:** a Department of Chemistry, School of Science, Payame Noor University (PNU), Ardakan, Yazd, Iran; b X-ray Crystallography Lab., Plasma Physics Research Center, Science and Research Branch, Islamic Azad University, Tehran, Iran; c Department of Chemistry, Science and Research Branch, Islamic Azad University, Tehran, Iran; d Department of Chemistry, Catalysis Division, University of Isfahan, Isfahan 81746-73441, Iran; e Department of Physics, University of Sargodha, Punjab, Pakistan

## Abstract

In the title compound, C_15_H_14_N_2_O_3_, the furan ring is disordered over two positions with a refined site-occupancy ratio of 0.587 (11):0.413 (11). The mean plane of the approximately planar pyrazole ring [maximum deviation = 0.0469 (11) Å] makes dihedral angles of 86.13 (11) and 4.5 (5)° with the phenyl and furan rings, respectively. The dihedral angle between the phenyl ring and the major component of the disordered furan ring is 81.8 (5)°. The mol­ecule shows chirality in one of the carbon atoms but the centrosymmetric space group means the compound is a racemic mixture. In the crystal, inter­molecular O—H⋯O and C—H⋯O hydrogen bonds connect the mol­ecules. The crystal structure is further stabilized by π–π stacking inter­actions with a centroid–centroid distance of 3.8646 (12) Å.

## Related literature

For standard bond lengths, see: Allen *et al.* (1987 ▶).
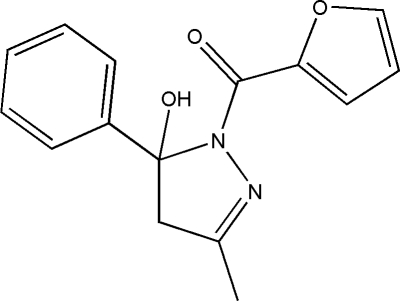


## Experimental

### 

#### Crystal data

C_15_H_14_N_2_O_3_
*M* *_r_* = 270.28
Monoclinic, 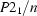

*a* = 10.6844 (10) Å
*b* = 8.4700 (7) Å
*c* = 15.3022 (16) Å
β = 95.266 (3)°
*V* = 1379.0 (2) Å^3^
*Z* = 4
Mo *K*α radiation
μ = 0.09 mm^−1^
*T* = 296 K
0.28 × 0.22 × 0.16 mm

#### Data collection

Bruker SMART APEXII CCD area-detector diffractometer
Absorption correction: multi-scan (*SADABS*; Bruker, 2005 ▶) *T*_min_ = 0.975, *T*_max_ = 0.985
10534 measured reflections
2495 independent reflections
1615 reflections with *I* > 2σ(*I*)
*R*_int_ = 0.044

#### Refinement

*R*[*F* ^2^ > 2σ(*F* ^2^)] = 0.042
*wR*(*F* ^2^) = 0.106
*S* = 1.03
2495 reflections
221 parameters
10 restraints
H-atom parameters constrained
Δρ_max_ = 0.12 e Å^−3^
Δρ_min_ = −0.13 e Å^−3^

### 

Data collection: *APEX2* (Bruker, 2005 ▶); cell refinement: *SAINT* (Bruker, 2005 ▶); data reduction: *SAINT*; program(s) used to solve structure: *SHELXTL* (Sheldrick, 2008 ▶); program(s) used to refine structure: *SHELXTL*; molecular graphics: *SHELXTL*; software used to prepare material for publication: *SHELXTL* and *PLATON* (Spek, 2009 ▶).

## Supplementary Material

Crystal structure: contains datablocks global, I. DOI: 10.1107/S1600536811000948/jh2253sup1.cif

Structure factors: contains datablocks I. DOI: 10.1107/S1600536811000948/jh2253Isup2.hkl

Additional supplementary materials:  crystallographic information; 3D view; checkCIF report

## Figures and Tables

**Table 1:** Hydrogen-bond geometry (Å, °)

*D*—H⋯*A*	*D*—H	H⋯*A*	*D*⋯*A*	*D*—H⋯*A*
O1—H1⋯O2^i^	0.82	2.02	2.7786 (18)	153
C14—H14*A*⋯O1^ii^	0.93	2.36	3.271 (9)	168

Symmetry codes: (i) 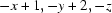; (ii) 
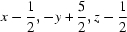
.

**Fig. 1.**
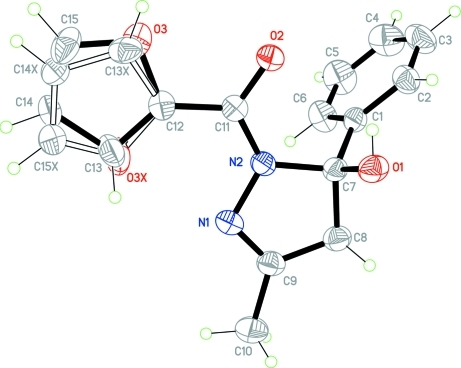
The molecular of the title compound, showing 40% probability displacement ellipsoids and the atomic numbering. The open bonds show the minor component of disordered furane ring.

**Fig. 2.**
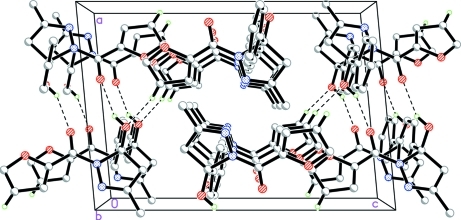
The packing of the compound viewed along the b-axis showing connecting of molecules through hydrogen bonds. All H atoms removed except those involved in the hydrogen bonds. Hydrogen bonds are shown as dashed lines.

## supplementary crystallographic information

### Comment

The asymmetric unit of the title compound, Fig. 1, comprises a substituted pyrazole. The furane ring shows flip-flop rotational disorder in two positions with refined site occupancy ratio of 0.587 (11)/0.413 (11). The mean plane of the approximately planar pyrazole ring [maximum deviation = 0.0469 (11)Å] makes dihedral angles of 86.13 (11) and 4.5 (5)° with phenyl and the major component of the furane rings, respectively. The dihedral angle between the phenyl ring and the major component of the disordered furane ring is 81.8 (5)°.

In the crystal structure, intermolecular O—H···O and C—H···O hydrogen bonds connect the components of the structure. The crystal structure is further stabilized by π–π stacking interactions [*Cg*1···*Cg*2^iii^ = 3.8646 (12)Å, (iii) x, y, z; *Cg*1 and *Cg*2 are the centroid of pyrazole and phenyl rings, respectively].

### Experimental

The title compound was synthesized by adding furan-2-carbohydrazide (2 mmol) to a solution of benzoylacetone (2 mmol) in ethanol (20 ml). The mixture was refluxed with stirring for half an hour. The resultant solution was filtered and the white single crystals suitable for *X*-ray structure determination were recrystallized from ethanol by slow evaporation of the solvents at room temperature over several days.

### Refinement

The H atoms of hydroxy group was located in a difference Fourier map and constraied to refine on the parent atom with U_iso_(H) = 1.5 U_eq_(O), see Table 1. The remaining H atoms were positioned geometrically with C—H = 0.93-0.97 Å and included in a riding-model approximation with U_iso_(H) = 1.2 or 1.5 U_eq_(C). A rotating group model was used for the methyl group. At first similarity and a series of distant restraints were applied to the furane rings but after refinement convereged, only the similarity restraints were removed.

### Crystal data

**Table:**

C_15_H_14_N_2_O_3_	*F*(000) = 568
*M**_r_* = 270.28	*D*_x_ = 1.302 Mg m^−^^3^
Monoclinic, *P*2_1_/*n*	Mo *K*α radiation, λ = 0.71073 Å
Hall symbol: -P 2yn	Cell parameters from 2245 reflections
*a* = 10.6844 (10) Å	θ = 2.5–29.5°
*b* = 8.4700 (7) Å	µ = 0.09 mm^−^^1^
*c* = 15.3022 (16) Å	*T* = 296 K
β = 95.266 (3)°	Block, white
*V* = 1379.0 (2) Å^3^	0.28 × 0.22 × 0.16 mm
*Z* = 4	

### Data collection

**Table:**

Bruker SMART APEXII CCD area-detector diffractometer	2495 independent reflections
Radiation source: fine-focus sealed tube	1615 reflections with *I* > 2σ(*I*)
graphite	*R*_int_ = 0.044
φ and ω scans	θ_max_ = 25.2°, θ_min_ = 2.4°
Absorption correction: multi-scan (*SADABS*; Bruker, 2005)	*h* = −12→12
*T*_min_ = 0.975, *T*_max_ = 0.985	*k* = −10→10
10534 measured reflections	*l* = −18→18

### Refinement

**Table:**

Refinement on *F*^2^	Secondary atom site location: difference Fourier map
Least-squares matrix: full	Hydrogen site location: inferred from neighbouring sites
*R*[*F*^2^ > 2σ(*F*^2^)] = 0.042	H-atom parameters constrained
*wR*(*F*^2^) = 0.106	*w* = 1/[σ^2^(*F*_o_^2^) + (0.037*P*)^2^ + 0.1913*P*] where *P* = (*F*_o_^2^ + 2*F*_c_^2^)/3
*S* = 1.03	(Δ/σ)_max_ < 0.001
2495 reflections	Δρ_max_ = 0.12 e Å^−^^3^
221 parameters	Δρ_min_ = −0.13 e Å^−^^3^
10 restraints	Extinction correction: *SHELXTL* (Sheldrick, 2008), Fc^*^=kFc[1+0.001xFc^2^λ^3^/sin(2θ)]^-1/4^
Primary atom site location: structure-invariant direct methods	Extinction coefficient: 0.0132 (14)

### Special details

**Table:**

Geometry. All esds (except the esd in the dihedral angle between two l.s. planes) are estimated using the full covariance matrix. The cell esds are taken into account individually in the estimation of esds in distances, angles and torsion angles; correlations between esds in cell parameters are only used when they are defined by crystal symmetry. An approximate (isotropic) treatment of cell esds is used for estimating esds involving l.s. planes.
Refinement. Refinement of F^2^ against ALL reflections. The weighted R-factor wR and goodness of fit S are based on F^2^, conventional R-factors R are based on F, with F set to zero for negative F^2^. The threshold expression of F^2^ > 2sigma(F^2^) is used only for calculating R-factors(gt) etc. and is not relevant to the choice of reflections for refinement. R-factors based on F^2^ are statistically about twice as large as those based on F, and R- factors based on ALL data will be even larger.

### Fractional atomic coordinates and isotropic or equivalent isotropic displacement parameters (Å^2^)

**Table:**

	*x*	*y*	*z*	*U*_iso_*/*U*_eq_	Occ. (<1)
O1	0.39871 (12)	1.05521 (16)	0.13086 (9)	0.0564 (4)	
H1	0.4501	1.0373	0.0954	0.085*	
O2	0.37249 (12)	0.97468 (15)	−0.05954 (9)	0.0540 (4)	
N1	0.11933 (14)	1.14352 (17)	0.03952 (12)	0.0530 (5)	
N2	0.22305 (14)	1.04995 (17)	0.02389 (10)	0.0463 (4)	
C1	0.30125 (18)	0.8002 (2)	0.09202 (12)	0.0467 (5)	
C2	0.4147 (2)	0.7260 (2)	0.10936 (14)	0.0605 (6)	
H2	0.4856	0.7842	0.1289	0.073*	
C3	0.4242 (3)	0.5637 (3)	0.09779 (18)	0.0803 (8)	
H3	0.5016	0.5142	0.1091	0.096*	
C4	0.3208 (3)	0.4768 (3)	0.0700 (2)	0.0916 (9)	
H4	0.3272	0.3682	0.0630	0.110*	
C5	0.2085 (3)	0.5500 (3)	0.0528 (2)	0.0909 (9)	
H5	0.1378	0.4910	0.0337	0.109*	
C6	0.1979 (2)	0.7105 (2)	0.06307 (16)	0.0704 (7)	
H6	0.1203	0.7591	0.0504	0.085*	
C7	0.28639 (17)	0.9765 (2)	0.10487 (13)	0.0455 (5)	
C8	0.19276 (18)	1.0218 (2)	0.17109 (14)	0.0569 (6)	
H8A	0.1500	0.9292	0.1909	0.068*	
H8B	0.2350	1.0751	0.2216	0.068*	
C9	0.10301 (18)	1.1294 (2)	0.12079 (15)	0.0545 (5)	
C10	0.0004 (2)	1.2167 (3)	0.15998 (16)	0.0818 (8)	
H10A	−0.0483	1.1441	0.1912	0.123*	
H10B	−0.0530	1.2667	0.1142	0.123*	
H10C	0.0363	1.2955	0.1998	0.123*	
C11	0.27454 (17)	1.0491 (2)	−0.05355 (13)	0.0436 (5)	
C12	0.21378 (16)	1.1311 (2)	−0.12971 (12)	0.0497 (5)	
C13	0.1182 (12)	1.2333 (18)	−0.1440 (6)	0.071 (3)	0.587 (11)
H13A	0.0724	1.2811	−0.1024	0.085*	0.587 (11)
C14	0.1026 (12)	1.2525 (13)	−0.2367 (6)	0.071 (3)	0.587 (11)
H14A	0.0360	1.3042	−0.2680	0.086*	0.587 (11)
C15	0.1996 (12)	1.1838 (19)	−0.2715 (7)	0.076 (3)	0.587 (11)
H15A	0.2191	1.1926	−0.3293	0.092*	0.587 (11)
O3	0.2646 (7)	1.0983 (13)	−0.2065 (3)	0.072 (2)	0.587 (11)
C13X	0.2557 (15)	1.136 (2)	−0.2096 (5)	0.060 (4)	0.413 (11)
H13B	0.3305	1.0955	−0.2267	0.072*	0.413 (11)
C14X	0.1599 (14)	1.2180 (19)	−0.2623 (9)	0.058 (3)	0.413 (11)
H14B	0.1533	1.2220	−0.3233	0.070*	0.413 (11)
C15X	0.0807 (19)	1.2883 (19)	−0.2115 (8)	0.079 (4)	0.413 (11)
H15B	0.0241	1.3695	−0.2263	0.095*	0.413 (11)
O3X	0.1028 (10)	1.212 (2)	−0.1327 (7)	0.078 (4)	0.413 (11)

### Atomic displacement parameters (Å^2^)

**Table:**

	*U*^11^	*U*^22^	*U*^33^	*U*^12^	*U*^13^	*U*^23^
O1	0.0494 (8)	0.0629 (8)	0.0569 (10)	−0.0070 (7)	0.0048 (7)	−0.0151 (7)
O2	0.0469 (8)	0.0640 (9)	0.0518 (9)	0.0075 (7)	0.0087 (7)	0.0015 (7)
N1	0.0495 (10)	0.0503 (9)	0.0598 (12)	0.0080 (8)	0.0091 (9)	−0.0036 (8)
N2	0.0456 (9)	0.0460 (9)	0.0478 (10)	0.0066 (7)	0.0073 (8)	0.0019 (7)
C1	0.0492 (12)	0.0482 (11)	0.0430 (12)	0.0013 (9)	0.0049 (9)	0.0042 (9)
C2	0.0586 (14)	0.0577 (13)	0.0655 (15)	0.0065 (11)	0.0072 (11)	0.0096 (11)
C3	0.0848 (18)	0.0643 (15)	0.094 (2)	0.0285 (14)	0.0197 (15)	0.0189 (14)
C4	0.117 (2)	0.0473 (14)	0.112 (2)	0.0000 (16)	0.020 (2)	0.0009 (14)
C5	0.095 (2)	0.0573 (15)	0.118 (3)	−0.0145 (14)	−0.0025 (18)	−0.0073 (15)
C6	0.0640 (15)	0.0533 (13)	0.0916 (19)	−0.0053 (11)	−0.0059 (13)	−0.0008 (12)
C7	0.0431 (11)	0.0502 (11)	0.0433 (12)	−0.0004 (9)	0.0034 (9)	−0.0034 (9)
C8	0.0566 (13)	0.0637 (13)	0.0514 (13)	0.0015 (10)	0.0109 (10)	−0.0045 (10)
C9	0.0485 (12)	0.0564 (12)	0.0592 (15)	0.0021 (10)	0.0083 (11)	−0.0080 (11)
C10	0.0691 (16)	0.1043 (19)	0.0746 (18)	0.0238 (14)	0.0214 (13)	−0.0108 (14)
C11	0.0408 (11)	0.0404 (10)	0.0495 (13)	−0.0054 (9)	0.0034 (9)	−0.0008 (9)
C12	0.0458 (12)	0.0492 (12)	0.0540 (14)	−0.0057 (10)	0.0041 (10)	0.0066 (10)
C13	0.063 (5)	0.076 (5)	0.075 (7)	0.026 (4)	0.016 (4)	0.030 (5)
C14	0.070 (7)	0.082 (6)	0.060 (6)	0.034 (5)	−0.006 (5)	0.013 (6)
C15	0.062 (7)	0.118 (10)	0.050 (4)	−0.013 (4)	0.009 (4)	0.022 (5)
O3	0.075 (3)	0.082 (6)	0.057 (3)	−0.007 (2)	−0.001 (2)	0.0081 (18)
C13X	0.075 (7)	0.050 (6)	0.061 (8)	0.000 (4)	0.032 (7)	0.006 (4)
C14X	0.062 (12)	0.065 (8)	0.051 (6)	0.005 (7)	0.016 (5)	0.011 (5)
C15X	0.106 (9)	0.073 (7)	0.059 (7)	0.033 (5)	0.009 (6)	0.010 (6)
O3X	0.066 (5)	0.100 (7)	0.067 (5)	0.015 (4)	0.003 (4)	0.038 (4)

### Geometric parameters (Å, °)

**Table:**

O1—C7	1.398 (2)	C8—H8B	0.9700
O1—H1	0.8200	C9—C10	1.493 (3)
O2—C11	1.232 (2)	C10—H10A	0.9600
N1—C9	1.277 (2)	C10—H10B	0.9600
N1—N2	1.401 (2)	C10—H10C	0.9600
N2—C11	1.352 (2)	C11—C12	1.457 (2)
N2—C7	1.492 (2)	C12—C13X	1.3407 (11)
C1—C2	1.370 (3)	C12—C13	1.3407 (10)
C1—C6	1.380 (3)	C12—O3	1.3670 (10)
C1—C7	1.516 (3)	C12—O3X	1.3677 (10)
C2—C3	1.390 (3)	C13—C14	1.4230 (11)
C2—H2	0.9300	C13—H13A	0.9300
C3—C4	1.363 (3)	C14—C15	1.3407 (10)
C3—H3	0.9300	C14—H14A	0.9300
C4—C5	1.355 (4)	C15—O3	1.3679 (10)
C4—H4	0.9300	C15—H15A	0.9300
C5—C6	1.374 (3)	C13X—C14X	1.4230 (10)
C5—H5	0.9300	C13X—H13B	0.9300
C6—H6	0.9300	C14X—C15X	1.3406 (10)
C7—C8	1.536 (3)	C14X—H14B	0.9300
C8—C9	1.486 (3)	C15X—O3X	1.3679 (10)
C8—H8A	0.9700	C15X—H15B	0.9300
			
C7—O1—H1	109.5	C9—C10—H10A	109.5
C9—N1—N2	107.11 (16)	C9—C10—H10B	109.5
C11—N2—N1	123.00 (16)	H10A—C10—H10B	109.5
C11—N2—C7	122.41 (15)	C9—C10—H10C	109.5
N1—N2—C7	113.55 (15)	H10A—C10—H10C	109.5
C2—C1—C6	118.50 (19)	H10B—C10—H10C	109.5
C2—C1—C7	121.90 (18)	O2—C11—N2	118.98 (17)
C6—C1—C7	119.60 (17)	O2—C11—C12	120.23 (17)
C1—C2—C3	120.2 (2)	N2—C11—C12	120.77 (16)
C1—C2—H2	119.9	C13X—C12—C13	98.3 (7)
C3—C2—H2	119.9	C13—C12—O3	110.4 (5)
C4—C3—C2	120.5 (2)	C13X—C12—O3X	108.3 (7)
C4—C3—H3	119.8	O3—C12—O3X	119.0 (6)
C2—C3—H3	119.8	C13X—C12—C11	125.7 (6)
C5—C4—C3	119.5 (2)	C13—C12—C11	135.6 (4)
C5—C4—H4	120.3	O3—C12—C11	114.0 (4)
C3—C4—H4	120.3	O3X—C12—C11	126.0 (5)
C4—C5—C6	120.7 (2)	C12—C13—C14	104.7 (6)
C4—C5—H5	119.6	C12—C13—H13A	127.7
C6—C5—H5	119.6	C14—C13—H13A	127.7
C5—C6—C1	120.7 (2)	C15—C14—C13	108.8 (9)
C5—C6—H6	119.7	C15—C14—H14A	125.6
C1—C6—H6	119.7	C13—C14—H14A	125.6
O1—C7—N2	110.47 (15)	C14—C15—O3	107.5 (8)
O1—C7—C1	114.13 (15)	C14—C15—H15A	126.3
N2—C7—C1	110.34 (15)	O3—C15—H15A	126.3
O1—C7—C8	106.70 (15)	C12—O3—C15	107.6 (7)
N2—C7—C8	99.82 (14)	C12—C13X—C14X	104.4 (8)
C1—C7—C8	114.43 (16)	C12—C13X—H13B	127.8
C9—C8—C7	103.87 (17)	C14X—C13X—H13B	127.8
C9—C8—H8A	111.0	C15X—C14X—C13X	110.3 (13)
C7—C8—H8A	111.0	C15X—C14X—H14B	124.8
C9—C8—H8B	111.0	C13X—C14X—H14B	124.8
C7—C8—H8B	111.0	C14X—C15X—O3X	103.6 (11)
H8A—C8—H8B	109.0	C14X—C15X—H15B	128.2
N1—C9—C8	114.99 (17)	O3X—C15X—H15B	128.2
N1—C9—C10	121.0 (2)	C12—O3X—C15X	110.2 (10)
C8—C9—C10	124.0 (2)		
			
C9—N1—N2—C11	−173.27 (17)	C7—N2—C11—C12	−175.89 (16)
C9—N1—N2—C7	−4.7 (2)	O2—C11—C12—C13X	−0.8 (13)
C6—C1—C2—C3	0.0 (3)	N2—C11—C12—C13X	−179.1 (12)
C7—C1—C2—C3	179.19 (19)	O2—C11—C12—C13	−171.2 (12)
C1—C2—C3—C4	−0.7 (4)	N2—C11—C12—C13	10.5 (13)
C2—C3—C4—C5	0.7 (4)	O2—C11—C12—O3	8.7 (6)
C3—C4—C5—C6	−0.1 (4)	N2—C11—C12—O3	−169.6 (5)
C4—C5—C6—C1	−0.6 (4)	O2—C11—C12—O3X	177.3 (10)
C2—C1—C6—C5	0.6 (3)	N2—C11—C12—O3X	−1.0 (11)
C7—C1—C6—C5	−178.6 (2)	C13X—C12—C13—C14	13.4 (15)
C11—N2—C7—O1	64.3 (2)	O3—C12—C13—C14	5.6 (16)
N1—N2—C7—O1	−104.43 (16)	O3X—C12—C13—C14	−128 (6)
C11—N2—C7—C1	−62.9 (2)	C11—C12—C13—C14	−174.5 (6)
N1—N2—C7—C1	128.44 (15)	C12—C13—C14—C15	−9.9 (18)
C11—N2—C7—C8	176.34 (16)	C13—C14—C15—O3	10.3 (19)
N1—N2—C7—C8	7.66 (18)	C13X—C12—O3—C15	−33 (5)
C2—C1—C7—O1	3.3 (3)	C13—C12—O3—C15	0.5 (15)
C6—C1—C7—O1	−177.48 (18)	O3X—C12—O3—C15	11.1 (13)
C2—C1—C7—N2	128.39 (19)	C11—C12—O3—C15	−179.5 (9)
C6—C1—C7—N2	−52.4 (2)	C14—C15—O3—C12	−6.8 (17)
C2—C1—C7—C8	−120.0 (2)	C13—C12—C13X—C14X	−11.5 (17)
C6—C1—C7—C8	59.2 (2)	O3—C12—C13X—C14X	137 (6)
O1—C7—C8—C9	107.68 (17)	O3X—C12—C13X—C14X	−3(2)
N2—C7—C8—C9	−7.31 (18)	C11—C12—C13X—C14X	175.3 (9)
C1—C7—C8—C9	−125.09 (17)	C12—C13X—C14X—C15X	14 (2)
N2—N1—C9—C8	−0.9 (2)	C13X—C14X—C15X—O3X	−18 (2)
N2—N1—C9—C10	179.00 (18)	C13X—C12—O3X—C15X	−8(2)
C7—C8—C9—N1	5.7 (2)	C13—C12—O3X—C15X	32 (5)
C7—C8—C9—C10	−174.22 (19)	O3—C12—O3X—C15X	−18.4 (19)
N1—N2—C11—O2	173.43 (16)	C11—C12—O3X—C15X	173.5 (11)
C7—N2—C11—O2	5.8 (2)	C14X—C15X—O3X—C12	16 (2)
N1—N2—C11—C12	−8.3 (3)		

### Hydrogen-bond geometry (Å, °)

**Table:**

*D*—H···*A*	*D*—H	H···*A*	*D*···*A*	*D*—H···*A*
O1—H1···O2^i^	0.82	2.02	2.7786 (18)	153
C14—H14A···O1^ii^	0.93	2.36	3.271 (9)	168

Symmetry codes: (i) −*x*+1, −*y*+2, −*z*; (ii) *x*−1/2, −*y*+5/2, *z*−1/2.
